# Non-Hermitian topology in rock–paper–scissors games

**DOI:** 10.1038/s41598-021-04178-8

**Published:** 2022-01-12

**Authors:** Tsuneya Yoshida, Tomonari Mizoguchi, Yasuhiro Hatsugai

**Affiliations:** Department of Physics, University of Tsukuba, Tsukuba, Ibaraki 305-8571 Japan

**Keywords:** Topological insulators, Evolution, Biological physics

## Abstract

Non-Hermitian topology is a recent hot topic in condensed matters. In this paper, we propose a novel platform drawing interdisciplinary attention: rock–paper–scissors (RPS) cycles described by the evolutionary game theory. Specifically, we demonstrate the emergence of an exceptional point and a skin effect by analyzing topological properties of their payoff matrix. Furthermore, we discover striking dynamical properties in an RPS chain: the directive propagation of the population density in the bulk and the enhancement of the population density only around the right edge. Our results open new avenues of the non-Hermitian topology and the evolutionary game theory.

## Introduction

In these decades, the notion of the topology has played a central role in condensed matter physics^1–5^. Analysis of topological aspects of condensed matters dates back to integer quantum Hall systems^6^ which exhibit robust chiral edge modes induced by the topology of the Hermitian Hamiltonian in the bulk^7–9^. While these robust edge modes are originally reported for quantum systems, they have been extended to various disciplines of science^10–20^. In particular, the notion of topology has been extended to an interdisciplinary field^21,22^; the emergence of topological edge modes has been predicted^22^ for networks of rock–paper–scissors (RPS) cycles which are described by the evolutionary game theory^23–28^.

Among the variety of topological phenomena, non-Hermitian topology^29–34^ has become one of the recent hot topics because it results in novel phenomena which do not have Hermitian counterparts. A representative example is the emergence of exceptional points (EPs)^35–38^ and their symmetry-protected variants^39–45^ on which eigenvalues of the non-Hermitian Hamiltonian touch both for the real- and imaginary-parts. Another typical example is a non-Hermitian skin effect^46–52^ which is extreme sensitivity of the eigenvalues and eigenstates to the presence/absence of boundaries. As is the case of the Hermitian topology, the above non-Hermitian phenomena have also been reported in a variety of systems^53–62^ (e.g., photonic systems^53–56^ and quantum systems^57–60,63^).

Despite the above significant progress, topological phenomena of the evolutionary game theory, which attract interdisciplinary attention, are restricted to the Hermitian topology. Highlighting non-Hermitian topology of such systems is significant as it may provide a new insights and may open a new avenue of the evolutionary game theory.

In this paper, we report non-Hermitian topological phenomena in the evolutionary game theory: an EP and a skin effect in RPS cycles. The EP in the single RPS cycle is protected by the realness of the payoffs which is mathematically equivalent to parity-time (*PT*) symmetry. Our linearized replicator equation elucidates that the EP governs dynamics of the RPS cycle. Furthermore, we discover striking dynamical phenomena in an RPS chain induced by the skin effect: the directive propagation of the population density in the bulk and the enhancement of the population density only around the right edge. These dynamical properties are in sharp contrast to those in the Hermitian systems. The above results open new avenues of the non-Hermitian topology and the evolutionary game theory.

## Results

### EP in a single RPS cycle

Firstly, we demonstrate the emergence of an EP at which two eigenvalues touch both for the real- and imaginary-parts.

**Figure 1:**
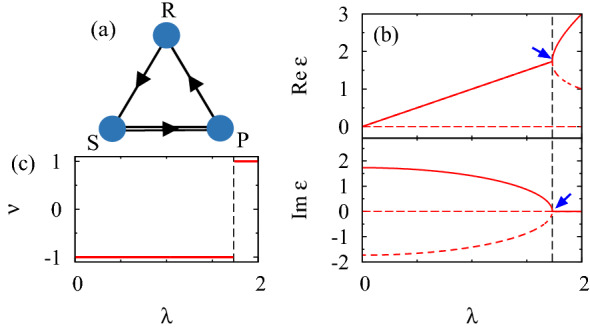
(**a**) Sketch of the RPS cycle. The arrows denote dominance relationship between the strategies for $\lambda =0$; “R” beats “S”; “S” beats “P”; “P” beats “R”. The second terms proportional to $\lambda$ are introduced between sites connected by the double line. (**b**) The real- and imaginary-parts of eigenvalues as functions of $\lambda$. An eigenvalue of $A(\lambda )$ is zero for an arbitrary $\lambda$ (see horizontal dashed lines). (**c**) The $\mathbb {Z}_2$-invariant characterizing the EP. The dashed vertical lines in panels (**b**,**c**) denote $\lambda =\lambda _{\mathrm {c}}$.

Consider two players play the RPS game (see Fig. 1a) whose payoff matrix is given by^64–67^

1 $$\begin{aligned} A(\lambda )= & {} \left( \begin{array}{ccc} 0 &{} -1 &{} 1 \\ 1 &{} 0 &{} -1 \\ -1 &{} 1 &{} 0 \end{array} \right) +\lambda \left( \begin{array}{ccc} 0 &{} 0 &{} 0 \\ 0 &{} 1 &{} -1 \\ 0 &{} -1 &{} 1 \end{array} \right) , \end{aligned}$$

with a real number $\lambda$. Here, players choose one of the strategies $(s_1,s_2,s_3)=(``\mathrm {R}",``\mathrm {P}",``\mathrm {S}")$. The payoff of a player is $A_{IJ}$ when the player chooses the strategy $s_I$ and the other player chooses $s_J$ ($I,J=1,2,3$). For $\lambda =0$, this game is reduced to the standard zero-sum RPS game where the sum of all players’ payoff is zero for an arbitrary set of strategies.

The above payoff matrix exhibits an EP, which can be deduced by noting the following two facts. (1) The first (second) term is anti-Hermitian (Hermitian). (2) For an arbitrary $\lambda$, eigenvalues $\epsilon _n$ ($n=1,2,3$) form a pair $\epsilon _n=\epsilon ^*_{n'}$ ($n\ne n'$) or are real numbers $\epsilon _n \in \mathbb {R}$. This constraint arises from the realness of the payoffs

2 $$\begin{aligned} \mathcal {K} A(\lambda ) \mathcal {K}= & {} A(\lambda ), \end{aligned}$$

where the operator $\mathcal {K}$ takes complex conjugate. Equation (2) can be recognized as *PT* symmetry by regarding $\lambda$ as a momentum (for more details, see Sect. S1 of Supplemental Material [68]). For $\lambda =0$, the energy eigenvalues are aligned along the imaginary axis due to the anti-Hermiticity of $A(\lambda =0)$. Increasing $\lambda$, two eigenvalues touch at a critical value $\lambda _\mathrm {c}$ so that they become real numbers when the second term is dominant. At $\lambda =\lambda _{\mathrm {c}}$, the EP emerges.

The emergence of the EP is supported by Fig. 1b. For $\lambda =0$, the energy eigenvalues are pure imaginary due to anti-Hermiticity of $A(\lambda =0)$. As $\lambda$ is turned on, two eigenvalues approach each other, and the EP emerges at $\lambda =\lambda _c=\sqrt{3}$. One can also characterize the topology of this EP by computing the $\mathbb {Z}_2$-invariant $\nu$^45^

3 $$\begin{aligned} \nu (\lambda )= & {} \mathrm {sgn}\left[ \mathrm {Disc}_E P(E,\lambda ) \right] , \end{aligned}$$

with $P(E,\lambda )=\mathrm {det}[A(\lambda )-E\mathbbm{1}]$. Here, $\mathrm {sgn}(x)$ takes 1 ($-1$) for $x>0$ ($x<0$), and $\mathrm {Disc}_E P(E,\lambda )$ denotes discriminant of $P(E,\lambda )$. For the $3\times 3$-matrix $A(\lambda )$, $\mathrm {Disc}_E P(E,\lambda )=(\epsilon _1-\epsilon _2)^2(\epsilon _1-\epsilon _3)^2(\epsilon _2-\epsilon _3)^2$ holds. Figure 1c plots the $\mathbb {Z}_2$-invariant as a function of $\lambda$. Corresponding to the emergence of the EP, the $\mathbb {Z}_2$-invariant jumps at $\lambda =\lambda _{\mathrm {c}}$, elucidating the topological protection of the EP.

### Dynamical properties and the EP

Suppose that a large number of players repeat the above game whose dynamics is described by the replicator equation [see Eq. (4)]. In this case, the EP governs the dynamical behaviors when the population density slightly deviates from a fixed point; the population density shows an oscillatory behavior for $0\le \lambda <\lambda _{\mathrm {c}}$, while such a behavior disappears for $\lambda _{\mathrm {c}}<\lambda$ due to the emergence of the EP.

Firstly, we linearize the replicator equation. When a large number of players repeat the game, the time-evolution is described by the replicator equation

4 $$\begin{aligned} \partial _t \varvec{e}^T_I \cdot \varvec{x}= \varvec{e}^T_I \cdot \varvec{x} \left( \varvec{e}^T_I A \varvec{x} -\varvec{x}^TA\varvec{x} \right) , \end{aligned}$$

where $\varvec{x}$ denotes the population density ($\sum _I x_I=1$), and $\varvec{e}_I$ is a unit vector whose *I*-th element is unity. The second term vanishes when the payoff matrix *A* is anti-symmetric. However, in order to access the non-Hermitian topology, the second term is inevitable which makes the argument in Ref.^22^ unavailable.

Nevertheless, we can still obtain the following linearized equation

5 $$\begin{aligned} \partial _t \delta \varvec{x}= \frac{1}{N_0} A \delta \varvec{x}, \end{aligned}$$

which is mathematically equivalent to the Schrödinger equation. This mathematical equivalence reveals that the dynamics of the RPS cycle (i.e., a classical system) can be understood in terms of quantum physics. Here, $\delta \varvec{x}$ is defined as $\delta \varvec{x}=\varvec{x}-\varvec{c}$ with $\varvec{c}=(1,1,\ldots ,1)^T/N_0$, and $N_0$ denotes dimensions of the matrix *A*. Key ingredients are the following relations:

6 $$\begin{aligned} \sum _{J}A_{IJ}=0 \quad&\mathrm {and}&\quad \sum _{J}A_{JI}=0, \end{aligned}$$

for an arbitrary *I*. This equation guarantees that $\varvec{c}$ denotes a fixed point. Linearizing the replicator equation around this fixed point, we obtain Eq. (5) as detailed in the “Methods”.

**Figure 2:**
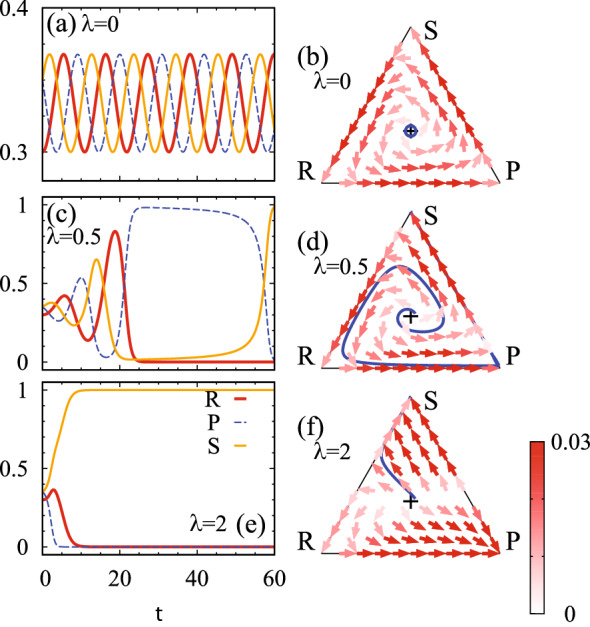
Dynamical properties of the RPS game (1). Panels (**a**,**c**,**e**) display the time-evolution of the population $\varvec{x}$ for $\lambda =0$, 0.5, and 2, respectively. The data are obtained by employing a fourth order Runge-Kutta method^68^ with discretized time $t_n=n\Delta t_{\mathrm {RK}}$ with $\Delta t_{\mathrm {RK}}=0.05$. We set the initial condition as $\varvec{x}(t=0)=(1-\delta _0,1+\delta _0/2,1+\delta _0/2)/3$ with $\delta _0=0.1$. The time-evolution is computed up to $t_n=120$. Panels (**b**,**d**,**f**) are phase plots for $\varvec{x}$. The arrows denote the direction of the dynamics $(\Delta x_1,\Delta x_2,\Delta x_3)$ which is computed by $\Delta x_I=x_I (\varvec{e}^T_IA\varvec{x}-\varvec{x}^TA\varvec{x})\Delta t$ with $\Delta t=0.1$. The black crosses denote the fixed point specified by $\varvec{c}=(1,1,1)/3$. In panels (**b**,**d**,**f**), the blue line denotes the population density $\varvec{x}(t)$ plotted in panels (**a**,**c**,**e**), respectively.

The linearized replicator equation indicates that the dynamics is governed by the spectrum of *A*. In particular, the imaginary-part of the eigenvalues results in oscillatory behaviors.

To verify the above statement, we numerically solve the replicator Eq. (4). Figure 2 plots the time-evolution of the population density for several values of $\lambda$. For $\lambda =0$, the matrix $A(\lambda )$ is reduced to the payoff matrix of the standard zero-sum RPS game. In this case, the eigenvalues of *A* are pure imaginary, which results in the oscillatory behavior (see Fig. 2a). This oscillatory behavior is also observed in the phase plot (see Fig. 2b), where the orbit forms a closed loop. The above oscillatory behavior is also observed for $\lambda =0.5$ (see Fig. 2c,d), which is due to the imaginary-part of the eigenvalues. We also note that the deviation $\delta \varvec{x}$ is enhanced as *t* increases, which is because the real-part of the eigenvalues is positive (see Fig. 1b). Further increasing $\lambda$ induces the EP (see Fig. 1b), and the eigenvalues become real. Correspondingly, the above oscillatory behavior is not observed for $\lambda =2$ (see Fig. 2e,f), The above numerical data verify that the EP governs the dynamics around the fixed point $\varvec{c}$; the oscillatory behavior of the RPS cycle disappears as the EP emerges. We note that for $\lambda < 0$, the fixed point corresponds to evolutionary stable strategy. A similar EP emerges also in this case which governs the dynamics.

We close this part with three remarks. Firstly, so far, we have seen the emergence of the EP in the RPS game by changing $\lambda$. We note that symmetry-protected exceptional rings are also observed by introducing an additional parameter (see Sect. S2 of Supplemental Material [68]), whose topology is also characterized by the $\mathbb {Z}_2$-invariant $\nu$. Secondly, although Refs.^69–72^ discuss topology of interaction networks among strategies (i.e., interaction topology), it differs from the topology discussed in this paper. Thirdly, we note that EPs are also reported for active matters^73,74^. We would like to stress, however, that significance of this paper is to reveal the emergence of the EPs and how they affect the dynamics in systems of the evolutionary game theory which describes the population density of biological systems and human societies.

### Skin effect in an RPS chain

Now, we discuss a one-dimensional system showing the skin effect whose origin is the non-trivial topology characterized by the winding number^48,49^. Because of this non-trivial topology, switching from the periodic boundary condition (PBC) to the open boundary condition (OBC) significantly changes the spectrum. Correspondingly, almost of all right eigenstates are localized around the right edge which are called skin modes.

**Figure 3:**
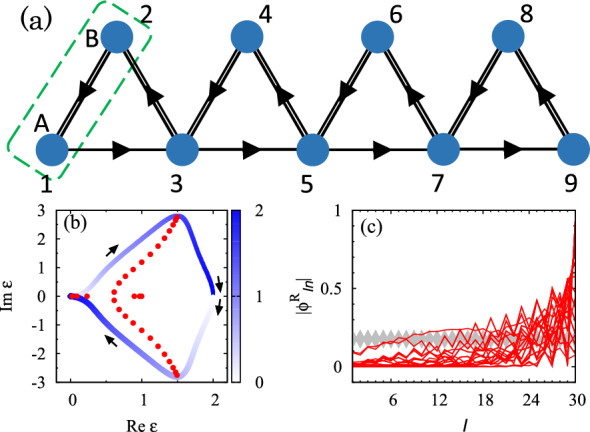
(**a**) Sketch of the RPS chain for $L_x=4$ with an additional site at $I=9$. The arrows describes payoffs. The terms proportional to $\lambda$ are introduced between sites connected by double lines. As shown in this panel, *I* takes $I=1,2,3,\ldots$. For the PBC, $I+2L_x=I$ holds. Dashed line denotes the unit cell. For $J=1,2,\ldots$, $R_{2I-1}= R_{2I}$ holds. (**b**) Spectrum of the RPS chain for $\lambda =-0.5$. The data colored with blue are obtained under the PBC. Here, the shade of color denotes $k/\pi$. The data denoted by red dots are obtained for $L_x=15$ and the OBC. (**c**) Amplitude of the right eigenvectors $\phi ^{\mathrm {R}}_{In}$ ($n=1,2,\ldots ,2L_x$) as functions of *I* for $L_x=15$. Red (gray) lines denote data for the OBC (PBC).

The above skin effect can be observed in an RPS chain illustrated in Fig. 3a. Applying the Fourier transformation, the payoff matrix is written as

7 $$\begin{aligned} A(k)= & {} \left( \begin{array}{cc} 0 &{} 1-e^{-ik} \\ -1+e^{ik} &{} -2i\sin k \end{array} \right) + \lambda \left( \begin{array}{cc} -2 &{} 1+e^{-ik} \\ 1+e^{ik} &{} -2 \end{array} \right) , \end{aligned}$$

under the PBC. Here, $\varvec{x}_{k}$ is defined as $\varvec{x}^T_{k}=(x_{k\mathrm {A}},x_{k\mathrm {B}})$ with $x_{k\alpha }=\frac{1}{L_x} \sum _{R_I} e^{ik R_I} x_{R_I\alpha }$ and $\alpha =\mathrm {A},\mathrm {B}$. Sets of $R_I$ and $\alpha _{I}$ are specified by *I* ($x_I=x_{R_I\alpha _I}$). For the explicit form of the payoff matrix in the real-space, see Sect. S3 of Supplemental Material [68].

Figure 3b plots the spectrum of the payoff matrix for $\lambda =-0.5$. When the PBC is imposed, eigenvalues form a loop structure as denoted by blue lines in Fig. 3b. Accordingly, the winding number^31,48,49^ defined as

8 $$\begin{aligned} W= & {} \oint \frac{dk}{2\pi i} \partial _k \log \mathrm {det}[A(k)-\epsilon _{\mathrm {ref}}\mathbbm{1} ], \end{aligned}$$

takes $-1$ for $\epsilon _{\mathrm {ref}}=1$, which implies the skin effect. Indeed, imposing the OBC significantly changes the spectrum (see red dots in Fig. 3b). Correspondingly, almost all of the right eigenvectors are localized around the edges, meaning the emergence of skin modes (see Fig. 3c).

**Figure 4:**
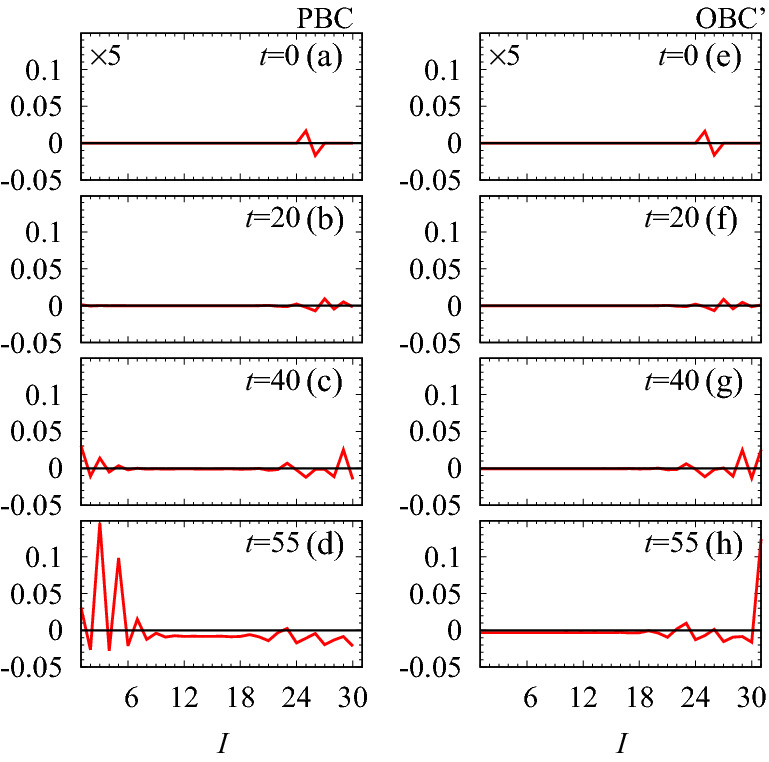
Time-evolution of the population density $\delta \varvec{x}(t)=\varvec{x}(t)-\varvec{c}$ for $\lambda =-0.5$ and $L_x=15$. The horizontal axis denotes *I*. (**a**–**f**) The time-evolution under the PBC [OBC’]. The data in panels (**a**,**e**) are multiplied by 5. The data are obtained by employing the fourth order Runge-Kutta method^68^ with discretized time $t_n=n\Delta t_{\mathrm {RK}}$ with $\Delta t_{\mathrm {RK}}=0.05$. We set the initial condition so that $N_0 x_{I}(0)$ takes $1+\delta _0$ and $1-\delta _0$ with $\delta _0=0.1$ for $I=25$ and $I=26$, respectively. For the other *I*, $N_0 x_{I}(0)$ takes 1. Here, $N_0$ is chosen so that $\sum _Ix_I(0)=1$ holds.

T﻿he above data (Fig. 3b,c) indicate that the skin effect is observed in the RPS chain. Here, we note that the spectrum and skin modes are almost unchanged under the following perturbation to the edges (see Sect. S3 of Supplemental Material [68]): attaching site at $I=2L_x+1$ and tuning diagonal elements to $A_{II}=\lambda$ only for $I=1,2L_x+1$ so that Eq. (6) holds (see Fig. 3a). We refer to this boundary condition as OBC’.

The above skin effect results in striking dynamical properties: the directive propagation of the population density in the bulk and the enhancement of the population density only around the right edge. The directive propagation is observed by imposing the PBC. Figure 4a–d indicate that the propagation only to the right direction is enhanced. This phenomenon can be understood by noting the following facts as well as linearized approximation: (1) as *t* increases, each mode is enhanced corresponding to $\mathrm {Re}\epsilon _n$; (2) the group velocity of each mode is proportional to $-\partial _k \mathrm {Im} \epsilon _n$. Because of the loop structure resulting in $W=-1$, the modes propagating to the right are more enhanced than the ones propagating to the left.

The enhancement of the population density at the right edge is observed by imposing OBC’. We note that Eq. (6) is satisfied for OBC’ while it is not for OBC. As shown in Fig. 4e–h, the population density around the right edge is enhanced as *t* increases. This phenomenon is due to the skin modes whose eigenvalues satisfy $\mathrm {Re}\epsilon _n>0$. The above dynamical behaviors are unique to non-Hermitian systems; turning off $\lambda$, $iA(\lambda =0)$ becomes Hermitian and the above behaviors disappear (see Sect. S3 of Supplemental Material [68]).

We close this part with two remarks. Firstly, we note that a similar behaviors can be observed for another RPS chain, implying ubiquity of the skin effect (see Sect. S4 of Supplemental Material [68]). Secondly, Ref.^21^ has analyzed an RPS chain whose payoff matrix is Hermitian up to the imaginary unit *i*. We would like to stress, however, that our aim is to observe non-Hermitian topological phenomena which are not accessible with the model in Ref.^21^. In this non-Hermitian case, we need to take into account the second term of Eq. (4).

### Discussion

We have proposed a new platform of non-Hermitian topology in an interdisciplinary field, i.e., RPS cycles described by the evolutionary game theory. Specifically, by analyzing the payoff matrix, we have demonstrated the emergence of the EP and the skin effect which are representative non-Hermitian topological phenomena. In addition, our linearized replicator equation has revealed that the EP governs the dynamics of the population density around the fixed point. Furthermore, we have discovered the striking dynamical phenomenon in the RPS chain: the directive propagation of the population density in the bulk and the enhancement of the population density only around the right edge which are induced by the skin effect.

Our results pose several future directions which we discuss below. The experimental observation is one of the central issues. In particular, our results provide a first step toward the observation of the non-Hermitian topology beyond natural science because the game theory describes a wide variety of systems from biological systems^25,75^ to human societies^26–28,76,77^; for instance, dynamics of human cooperation has been discussed in Refs.^76,77^. The experimental observation in such a system is considered to be a significant step to the application of topological phenomena beyond natural science. In addition, as is the case of equatorial wave^18^, our result may provide a novel perspective of well-known phenomena for biological systems such as bacteria^25^ and side-blotched lizards^75^; dynamics of such systems may be understood in terms of exceptional points. We also note that the topological classification for systems described by the game theory is also a crucial issue to be addressed.

## Methods

### Derivation of the linearized replicator equation

Here, we derive Eq. (5). We start with noting Eq. (6) results in the relations $A\varvec{c}=0$ and $\varvec{c}^TA=0$ which mean that $\varvec{c}=(1,1,1,\ldots ,1)^T/N_0$ is a fixed point. By making use of the above relations we have

9 $$\begin{aligned}&\partial _t \varvec{e}^T_I \cdot \delta \varvec{x} \nonumber \\&\quad = \varvec{e}^T_I \cdot (\varvec{c}+\delta \varvec{x}) \left[ \varvec{e}^T_I A (\varvec{c}+\delta \varvec{x}) -(\varvec{c}+\delta \varvec{x})^T A (\varvec{c}+\delta \varvec{x}) \right] \nonumber \\&\quad = \varvec{e}^T_I \cdot (\varvec{c}+\delta \varvec{x}) \left( \varvec{e}^T_I A \delta \varvec{x} -\delta \varvec{x}^TA \delta \varvec{x} \right) \nonumber \\&\quad \sim (\varvec{e}^T_I \cdot \varvec{c}) \varvec{e}^T_I A \delta \varvec{x}. \end{aligned}$$

From the second to the third line, we have used the relations $A\varvec{c}=0$ and $\varvec{c}A=0$. In the last line, we have discarded the second and third order terms of $\delta \varvec{x}$.

Because $(\varvec{e}^T_I \cdot \varvec{c})=1/N_0$ for an arbitrary *I*, we have

10 $$\begin{aligned} \partial _t \delta \varvec{e}^T_I\cdot \varvec{x}\sim & {} \frac{1}{N_0} \varvec{e}^T_I A \delta \varvec{x}, \end{aligned}$$

which is equivalent to Eq. (5).

## Supplementary Information

Supplementary Information.
