# Supplementary material for: Non-Hermitian topology in rock–paper–scissors games
Source: Sci Rep. 2022 Jan 12;12:560. doi: 10.1038/s41598-021-04178-8 (PMC8755820; doi:10.1038/s41598-021-04178-8)
Supplement: Supplementary file 1 — Supplementary Information. [file 41598_2021_4178_MOESM1_ESM.pdf]

Supplemental Materials:
Non-Hermitian topology in rock-paper-scissors games

S1. PT SYMMETRY

Here, we briefly review topological properties under PT symmetry (i.e., symmetry described by the product of the time-reversal operation and inversion). When the system is PT symmetric, the Bloch Hamiltonian $h(\boldsymbol{k})$ satisfies

$$PTh(\boldsymbol{k})PT^{-1} = h(\boldsymbol{k}), \tag{S1}$$

with an anti-unitary operator $PT = U_{PT}\mathcal{K}$, a unitary matrix U_{PT}, and $\boldsymbol{k}$ denoting the momentum. Here, each of the time-reversal operation and the spatial inversion flips the momentum ($\boldsymbol{k} \to -\boldsymbol{k}$), and thus, their product does not flip $\boldsymbol{k}$.

Equation (S1) results in the following constraint on the eigenvalue ϵ_n ($n = 1, 2, \ldots$)

$$\epsilon_n = \epsilon^*_{n'} \text{ or } \epsilon_n \in \mathbb{R}. \tag{S2}$$

The above equation is obtained by a straightforward calculation. Suppose that $|\psi_n(\boldsymbol{k})\rangle_R$ and $\epsilon_n(\boldsymbol{k})$ are a right eigenvector and an eigenvalue of $h(\boldsymbol{k})$, $h(\boldsymbol{k})|\psi_n(\boldsymbol{k})\rangle_R = \epsilon_n(\boldsymbol{k})|\psi_n(\boldsymbol{k})\rangle_R$. Then, $PT|\psi_n(\boldsymbol{k})\rangle_R$ is an eigenstate with eigenvalue ϵ^*_n because

$$\begin{aligned} h(\boldsymbol{k})PT|\psi_n(\boldsymbol{k})\rangle_R &= PTh(\boldsymbol{k})|\psi_n(\boldsymbol{k})\rangle_R \\ &= \epsilon^*_n(\boldsymbol{k})PT|\psi_n(\boldsymbol{k})\rangle_R, \end{aligned} \tag{S3}$$

holds. Therefore, we obtain Eq. (S2).

Now, let us discuss the topology for $PT^2 = 1$. When a point-gap opens at $\epsilon_{\mathrm{ref}} \in \mathbb{R}$ [i.e., $\det(h - \epsilon_{\mathrm{ref}}) \neq 0$ holds], the system may possess topological properties which are characterized by the following zero-dimensional $\mathbb{Z}_2$-invariant ν'

$$\nu' = -\mathrm{sgn}[\det(h - \epsilon_{\mathrm{ref}})], \tag{S4}$$

with $\mathrm{sgn}(x)$ takes 1 (-1) for $x > 0$ ($x < 0$). For $\epsilon_{\mathrm{ref}} = \epsilon_{\mathrm{EP}}$ ($\epsilon_{\mathrm{EP}} = \sqrt{3}$), ν' and ν defined in Eq. (3) characterizes the same topology of Eq. (1); because $\nu' = \prod_n(\epsilon_n - \epsilon_{\mathrm{ref}})$ holds, we have ν' taking 1 (-1) for $\lambda > \lambda_{\mathrm{c}}$ ($\lambda < \lambda_{\mathrm{c}}$).

S2. SYMMETRY-PROTECTED EXCEPTIONAL RINGS IN AN RPS CYCLE

We demonstrate the emergence of a symmetry-protected exceptional rings in evolutionary game theory. Consider an extended RPS cycle whose payoff matrix is written as

$$A(\lambda, \kappa) = A(\lambda) + \kappa \begin{pmatrix} 1 & 0 & -1 \\ 0 & 0 & 0 \\ -1 & 0 & 1 \end{pmatrix}, \tag{S5}$$

with $\lambda, \kappa \in \mathbb{R}$.

We can block-diagonalize the matrix A. Applying the unitary transformation with

$$V = \begin{pmatrix} \frac{1}{\sqrt{3}} & \frac{1}{\sqrt{2}} & \frac{1}{\sqrt{6}} \\ \frac{1}{\sqrt{3}} & -\frac{1}{\sqrt{2}} & \frac{1}{\sqrt{6}} \\ \frac{1}{\sqrt{3}} & 0 & \frac{-\sqrt{2}}{\sqrt{3}} \end{pmatrix}, \tag{S6}$$

we have

$$V^T A(\lambda, \kappa) V = \begin{pmatrix} 0 & 0 & 0 \\ 0 & 0 & -\sqrt{3} \\ 0 & \sqrt{3} & 0 \end{pmatrix} + \lambda \begin{pmatrix} 0 & 0 & 0 \\ 0 & \frac{1}{2} & -\frac{\sqrt{3}}{2} \\ 0 & -\frac{\sqrt{3}}{2} & \frac{3}{2} \end{pmatrix} + \kappa \begin{pmatrix} 0 & 0 & 0 \\ 0 & \frac{1}{2} & \frac{\sqrt{3}}{2} \\ 0 & \frac{\sqrt{3}}{2} & \frac{3}{2} \end{pmatrix}. \tag{S7}$$

Diagonalizing the above matrix, we have eigenvalues 0 and

$$\epsilon_\pm = (\lambda + \kappa) \pm \sqrt{\frac{3}{4}(\lambda - \kappa)^2 + \frac{1}{4}(\lambda + \kappa)^2 - 3}. \tag{S8}$$

We note that for $\kappa = 0$, the EP emerges at $\lambda = \lambda_c = \sqrt{3}$.

Figure S1(a) [S1(b)] plots the real- [imaginary] part of $\epsilon_{\pm}$. These figures indicate the emergence of the symmetry-protected exceptional rings; EPs, where the two bands touch both for the real- and the imaginary-parts, form a ring. We also note that ν takes -1 (1) inside (outside) of the ring, elucidating the topological protection.

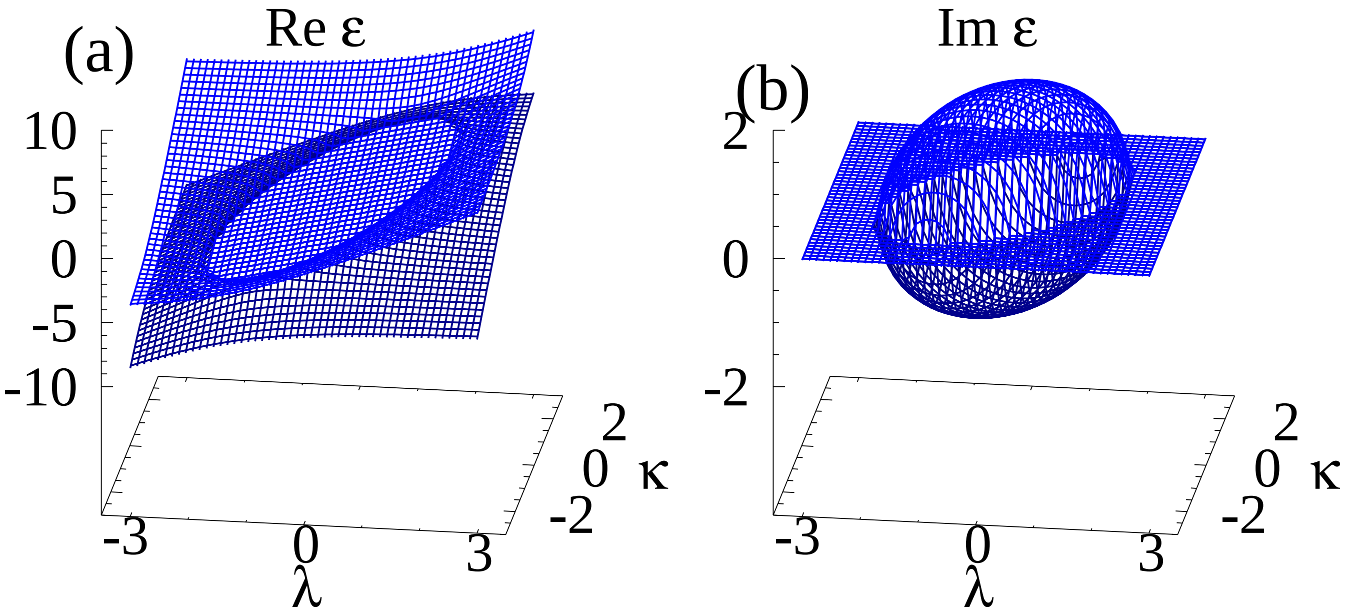

FIG. S1. (a) and (b): The real- and imaginary-parts of $\epsilon_{\pm}$. The data colored with blue (dark blue) represents ϵ_+ (ϵ_-).

The above results verify the emergence of the symmetry-protected exceptional rings in the extended RPS cycle.

S3. DETAILS OF THE RPS CHAIN

We discuss details of the RPS chain defined in the main text. Under the OBC', the payoff matrix is written as

$$A(\lambda) = A_0 + \lambda B. \tag{S9}$$

We have introduced an additional site at $I = 9$ [see Fig. 3(a)] for $L_x = 4$. Hence, the vector $\boldsymbol{x}$ consists of nine components, $\boldsymbol{x} = (x_1, x_2, x_3, \cdots, x_9)^T$. Here, A_0 and B are defined as

$$A_0 = \begin{pmatrix} A_{0\mathrm{d}} & A_{0\mathrm{c}}^T & 0 & 0 & 0 \\ A_{0\mathrm{c}} & A_{0\mathrm{d}} & A_{0\mathrm{c}}^T & 0 & 0 \\ 0 & A_{0\mathrm{c}} & A_{0\mathrm{d}} & A_{0\mathrm{c}}^T & 0 \\ 0 & 0 & A_{0\mathrm{c}} & A_{0\mathrm{d}} & A_{0\mathrm{cR}}^T \\ 0 & 0 & 0 & A_{0\mathrm{cR}} & 0 \end{pmatrix}, \tag{S10a}$$

with

$$A_{0\mathrm{d}} = \begin{pmatrix} 0 & 1 \\ -1 & 0 \end{pmatrix}, \quad A_{0\mathrm{c}} = \begin{pmatrix} -1 & 0 \\ 1 & 0 \end{pmatrix}, \quad A_{0\mathrm{cR}} = \begin{pmatrix} 1 & -1 \end{pmatrix}, \tag{S10b}$$

and

$$B = \begin{pmatrix} B_{\mathrm{dL}} & B_{\mathrm{c}}^T & 0 & 0 & 0 \\ B_{\mathrm{c}} & B_{\mathrm{d}} & B_{\mathrm{c}}^T & 0 & 0 \\ 0 & B_{\mathrm{c}} & B_{\mathrm{d}} & B_{\mathrm{c}}^T & 0 \\ 0 & 0 & B_{\mathrm{c}} & B_{\mathrm{d}}^T & B_{\mathrm{cR}}^T \\ 0 & 0 & 0 & B_{\mathrm{cR}}^T & -1 \end{pmatrix}, \tag{S11a}$$

with

$$B_{\rm d} = \begin{pmatrix} -2 & 1 \\ 1 & -2 \end{pmatrix}, \quad B_{\rm c} = \begin{pmatrix} 0 & 1 \\ 0 & 0 \end{pmatrix}, \quad B_{\rm dL} = \begin{pmatrix} -1 & 1 \\ 1 & -2 \end{pmatrix}, \quad B_{\rm cR} = \begin{pmatrix} 0 & 1 \end{pmatrix}, \tag{S11b}$$

respectively. In addition, we have imposed $B_{11} = B_{99} = -1$ to satisfy Eq. (6).

In Fig. S2, we can see that the spectrum and amplitude of the right eigenvectors for OBC' are similar to those for OBC, which indicates the robustness of the skin effect against perturbations. This is because the non-Hermitian topology induces the skin effect. We also note that $\boldsymbol{c} = (1, 1, \ldots, 1)/31$ is the right eigenvector for $L_x = 15$ [see Fig. S2(b)] because Eq. (6) holds under OBC'.

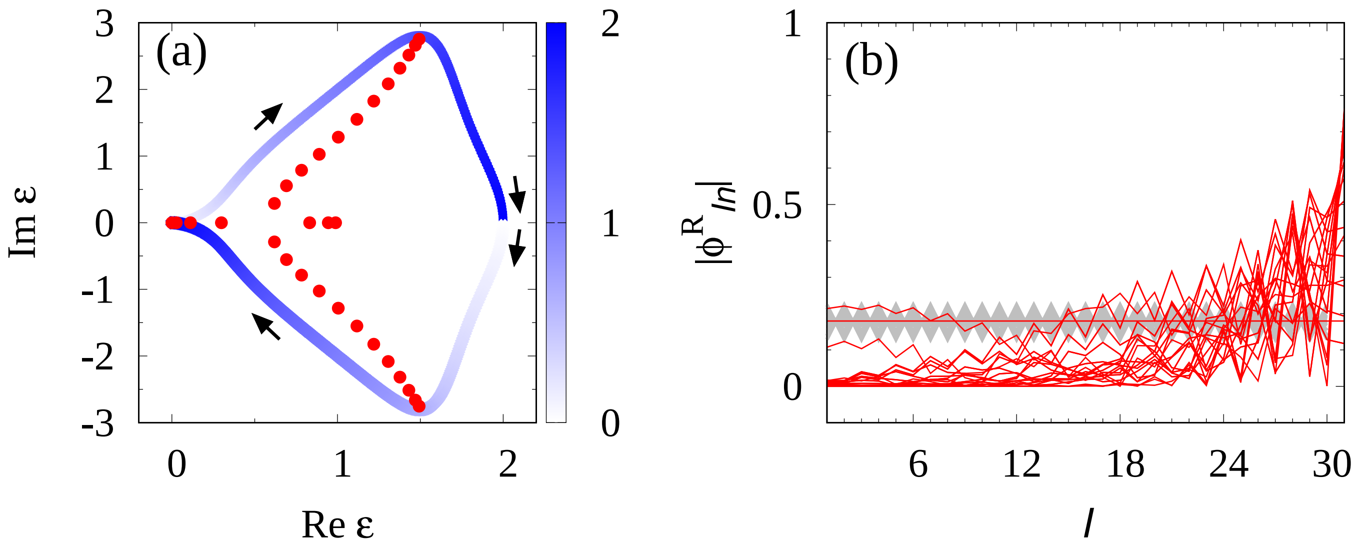

FIG. S2. Spectrum and amplitude of right eigenvectors under the OBC'. (a): The spectrum for $\lambda = -0.5$ and $L_x = 15$. (b):Amplitude of the right eigenvectors $\phi^{\rm R}_{In}$ $(n = 1, 2, \ldots, 2L_x + 1)$ as functions of I for $L_x = 15$. The data are plotted in a similar way to Fig. 3.

When Eq. (6) is satisfied, the replicator equation (4) can be linearized as discussed in the main text. In this case, the dynamics is governed by

$$i\partial_t \delta\boldsymbol{x}(t) = H\delta\boldsymbol{x}(t), \tag{S12}$$

with $H = iA/N_0$ [see also Eq. (5)] which is mathematically equivalent to the Schrödinger equation. In quantum systems, the group velocity and the lifetime are written as $\partial_k {\rm Re}E_n(k)$ and $1/{\rm Im}E_n(k)$ $({\rm Im}E_n(k) > 0)$, respectively. Here, $E_n(k)$ $(n = 1, 2, \ldots)$ denotes eigenvalues of H.

Figure S3 displays the dynamical properties for $\lambda = 0$ where the payoff matrix is anti-Hermitian. Because all eigenvalues ϵ's are pure imaginary, the directive perpetration which arises from the real-part of the eigenvalues ϵ's is not observed. In addition, no localized mode is enhanced due to the absence of skin modes.

S4. ANOTHER RPS CHAIN

We introduce another RPS chain [see Fig.S4(a)] exhibiting the skin effect. For $L_x = 5$, the payoff matrix is written as

$$A = \begin{pmatrix} A_0 & 0 & 0 & 0 & A'_{\rm c} \\ A_{\rm c} & A_0 & 0 & 0 & 0 \\ 0 & A_{\rm c} & A_0 & 0 & 0 \\ 0 & 0 & A_{\rm c} & A_0 & 0 \\ 0 & 0 & 0 & A_{\rm c} & A_0 \end{pmatrix}, \tag{S13a}$$

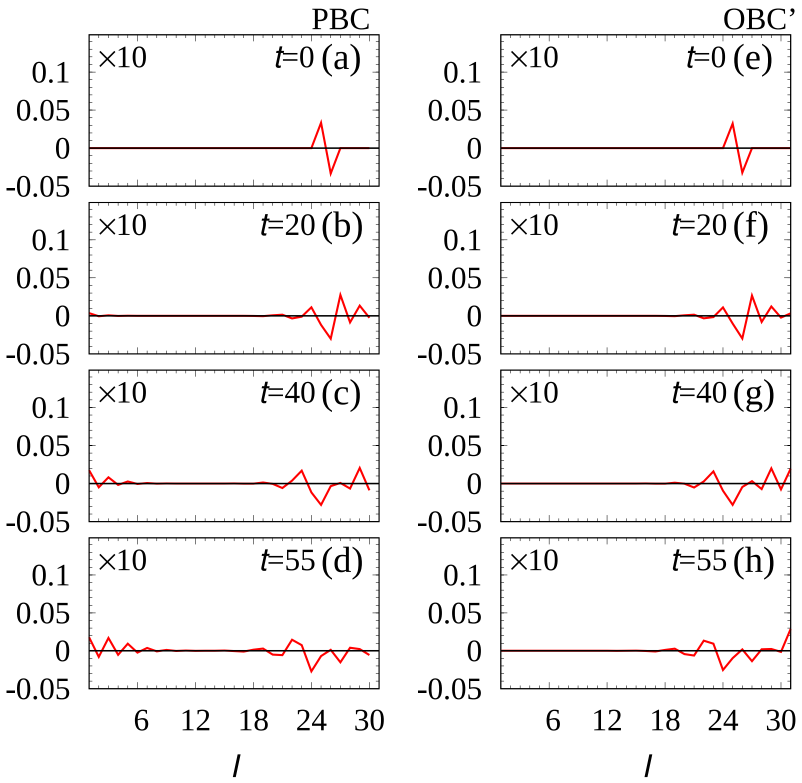

FIG. S3. Time-evolution of the population density $\delta \boldsymbol{x}(t) = \boldsymbol{x}(t) - \boldsymbol{c}$ for $\lambda = 0$ and $L_x = 15$. The horizontal axis denotes I. (a)-(d) [(e)-(f)]: The time-evolution under the PBC [OBC’]. The data in all panels are multiplied by 10. These data are obtained in the same way as those of Fig. 4.

with

$$A_0 = \begin{pmatrix} d & 0 & r_2 \\ r_3 & d & 0 \\ 0 & r_1 & d \end{pmatrix}, \tag{S13b}$$

$$A_{\mathrm{c}} = \begin{pmatrix} 0 & 0 & r_4 \\ 0 & 0 & 0 \\ 0 & 0 & 0 \end{pmatrix}, \tag{S13c}$$

and the vector $\boldsymbol{x}$ consists of fifteen components,

$$\boldsymbol{x} = (x_1, x_2, x_3, \cdots, x_{15})^T. \tag{S13d}$$

Here, A'_{c} is equal to A_{c} (the zero matrix) under the PBC (OBC).

Applying the Fourier transformation, the payoff matrix is written as

$$A(k) = \begin{pmatrix} d & 0 & r_2 + r_4 e^{ik} \\ r_3 & d & 0 \\ 0 & r_1 & d \end{pmatrix}, \tag{S14}$$

under the PBC. Here, $\boldsymbol{x}_k$ is defined as $\boldsymbol{x}_k^T = (x_{k\mathrm{S}}, x_{k\mathrm{R}}, x_{k\mathrm{P}})$ with $x_{ks} = \frac{1}{L_x} \sum_{R_I} e^{ikR_I} x_{R_I s}$ and $s = \mathrm{R}, \mathrm{P}, \mathrm{S}$. Sets of R_I and s_I are specified by I ($x_I = x_{R_I s_I}$).

Figure S4(b) plots the spectrum of the payoff matrix for $(r_1, r_2, r_3, r_4, d) = (1, 1/2, 1, 1/2, -1)$. When the PBC is imposed, eigenvalues form a loop structure as denoted by blue lines in Fig. S4(b). Accordingly, the winding number [Eq. (8)] takes 1 for $\epsilon_{\mathrm{ref}} = 0.5$, which implies the skin effect. Indeed, imposing the OBC significantly changes the spectrum [see red dots in Fig. S4(b)]. Correspondingly, all of the right eigenvectors are localized around the edges, meaning the emergence of skin modes [see Fig. S4(c)]. The above data [Figs. S4(b) and S4(c)] indicate that the skin effect is observed in this RPS chain as well. The essentially same results of eigenvalues and eigenvectors are

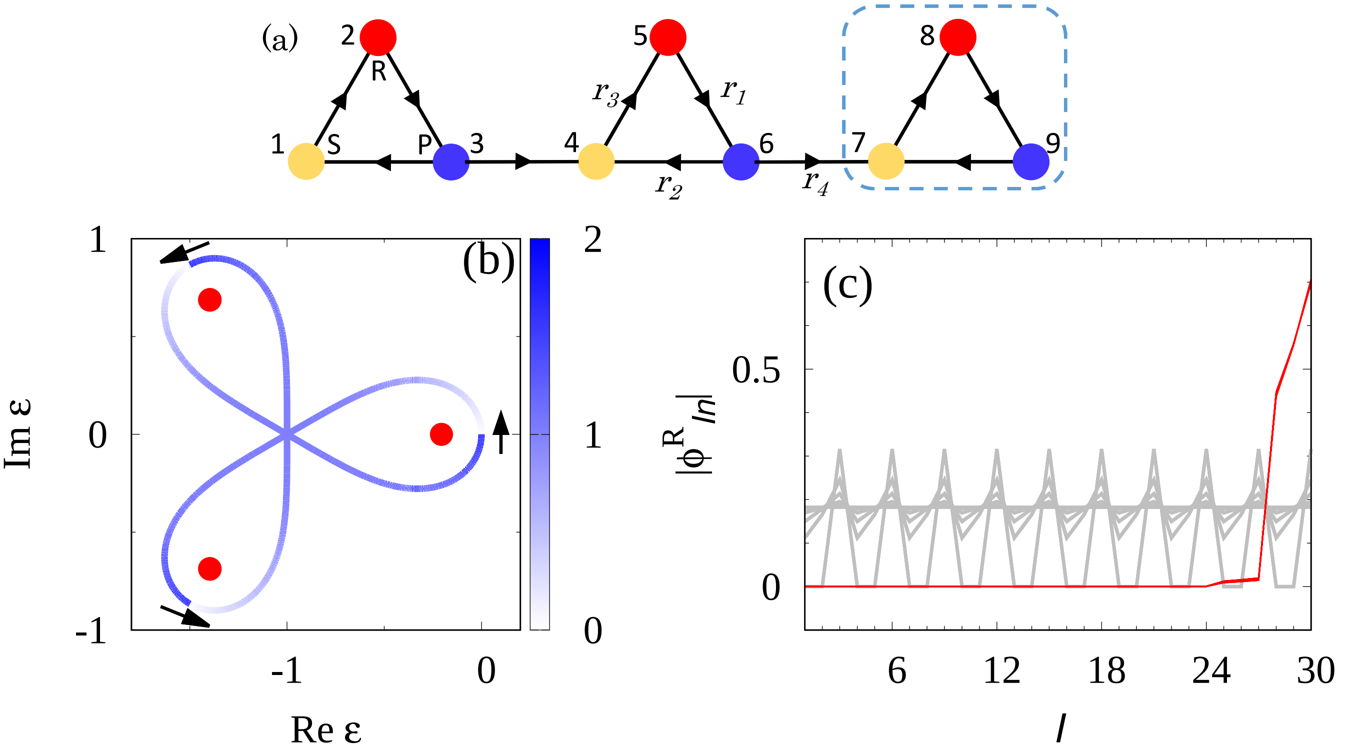

FIG. S4. (a): Sketch of the RPS chain for $L_x = 3$. The arrows and r's describe payoffs. Here, $A_{II} = d$ for an arbitrary I. As shown in this panel, I takes $I = 1, 2, \ldots, 3L_x$. For the PBC, $I + 3L_x = I$ holds. Dashed line denotes the unit cell. For $J = 1, 2, \ldots, L_x$, $R_{3J-2} = R_{3J-1} = R_{3J} = J$ holds. (b): Spectrum of the RPS chain for $(r_1, r_2, r_3, r_4, d) = (1, 1/2, 1, 1/2, -1)$. The data colored with blue are obtained under the PBC. Here, the shade of color denotes k/π. The data colored with red are obtained for $L_x = 10$ and the OBC. (c): Amplitude of the right eigenvectors $|\phi^{R}_{In}|$ $n = 1, 2, \ldots, 3L_x$ as functions of I for $L_x = 10$. Red (gray) lines denote data for the OBC (PBC). For the OBC, all of the eigenvectors are localized around the right edge.

also obtained for $(r_1, r_2, r_3, r_4, d) = (2, 2, 2, 2, 1)$. For this parameter set, the chain is composed of an ordinary RPS cycles [1] whose payoff matrix is written as $\begin{pmatrix} 1 & 0 & 2 \\ 2 & 1 & 0 \\ 0 & 2 & 1 \end{pmatrix}$.

As discussed in main text, the non-Hermitian topology characterized by $W = 1$ induces the directive propagation under the PBC [see Fig. S5(a)-S5(d)] where Eq. (6) is satisfied for this parameter set. We also note that under the OBC, the players prefer to be localized around the right edge for large t [see Fig. S5(e)-S5(f)]. However, introducing the edges violates Eq. (6) which prevents us from mapping Eq. (4) to the Schrödinger equation; introducing perturbations around the edge does not help to recover Eq. (6) for this model.

Z. Wang, B. Xu & H.-J. Zhou Social cycling and conditional responses in the rock-paper-scissors game. *Scientific Reports*, **4**, 5830 (2014).

E. Süli & D. F. Mayers *An introduction to numerical analysis*. Cambridge university press (2003).

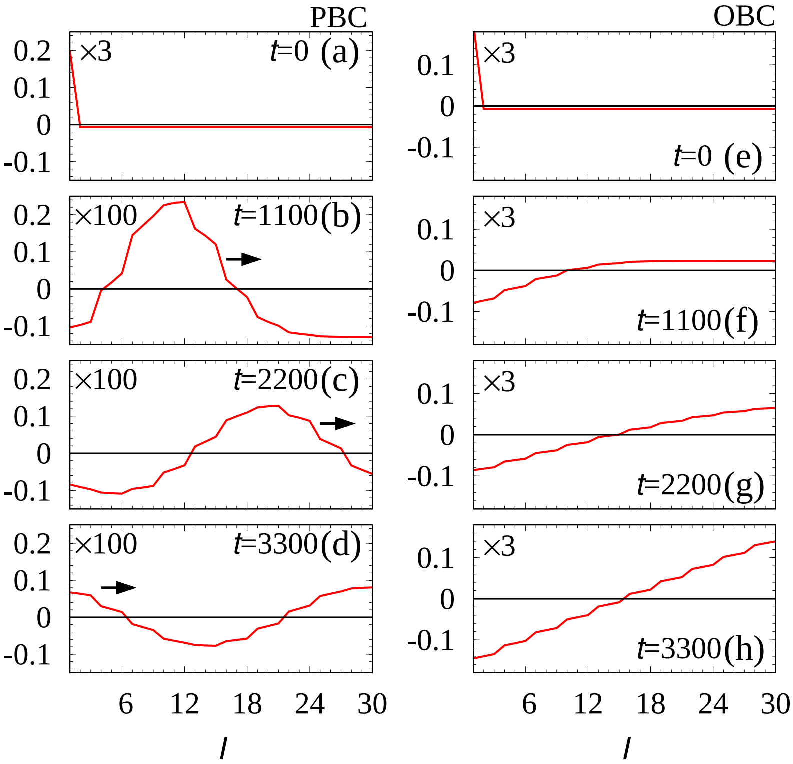

FIG. S5. The time-evolution of the population density $\delta\boldsymbol{x}(t) = \boldsymbol{x}(t) - \boldsymbol{c}$ for $(r_1, r_2, r_3, r_4, d) = (1, 1/2, 1, 1/2, -1)$ and $L_x = 10$. The horizontal axis denotes I. (a)-(d) [(e)-(f)]: The time-evolution under the PBC [OBC]. The data in panels (b)-(d) [(a) and (e)-(h)] are multiplied by 100 [3]. The data are obtained by employing the fourth order Runge-Kutta method [2] with discretized time $t_n = n\Delta t_{\mathrm{RK}}$ with $\Delta t_{\mathrm{RK}} = 0.05$. We set the initial condition as $\delta\boldsymbol{x}(0) = \frac{\delta_0}{(3L_x-1)3L_x}(3L_x - 1, -1, -1, \ldots, -1)$ with $\delta_0 = 2$.
